# Wellbeing among sub-Saharan African patients with advanced HIV and/or cancer: an international multicentred comparison study of two outcome measures

**DOI:** 10.1186/1477-7525-12-80

**Published:** 2014-05-31

**Authors:** Richard Harding, Lucy Selman, Zippy Ali, Richard A Powell, Eve Namisango, Faith Mwangi-Powell, Liz Gwyther, Nancy Gikaara, Irene J Higginson, Richard J Siegert

**Affiliations:** 1 Department of Palliative Care, Policy & Rehabilitation, King’s College London, Cicely Saunders Institute, Bessemer Road, London SE5 9PJ, UK; 2 Kenyan Hospice Palliative Care Association, Nairobi, Kenya; 3 Formerly African Palliative Care Association, Kampala, Uganda; 4 African Palliative Care Association, Kampala, Uganda; 5 Hospice Palliative Care Association of South Africa, School of Public Health and Family Medicine, University of Cape Town, Cape Town, South Africa; 6 Person Centred Research Centre, Faculty of Health and Environmental Sciences, Auckland University of Technology, Auckland, New Zealand

**Keywords:** Sub-Saharan Africa, HIV, Cancer, Palliative care, Outcome, Self-report

## Abstract

**Background:**

Despite the high mortality rates of HIV and cancer in sub-Saharan Africa, there are few outcome tools and no comparative data across conditions. This study aimed to measure multidimensional wellbeing among advanced HIV and/or cancer patients in three African countries, and determine the relationship between two validated outcome measures.

**Methods:**

Cross-sectional self-reported data from palliative care populations in Kenya, Uganda and South Africa using FACIT-G+Pal and POS measures.

**Results:**

Among 461 participants across all countries, subscale “social and family wellbeing” had highest (best) score. Significant country effect showed lower (worse) scores for Uganda on 3 FACIT G subscales: Physical, Social + family, and functional. In multiple regression, country and functional status accounted for 21% variance in FACIT-Pal. Worsening functional status was associated with poorer POS score. Kenyans had worse POS score, followed by Uganda and South Africa. Matrix of correlational coefficients revealed moderate correlation between the POS and FACIT-Pal core scale (0.60), the FACIT-G and POS (0.64), and FACIT-G + Pal with POS (0.66).

**Conclusions:**

The data reveal best status for family and social wellbeing, which may reflect the sample being from less individualistic societies. The tools appear to measure different constructs of wellbeing in palliative care, and reveal different levels of wellbeing between countries. Those with poorest physical function require greatest palliative and supportive care, and this does not appear to differ according to diagnosis.

## Background

In 2012 an estimated 25 million people in sub-Saharan Africa lived with HIV infection and there were 1.2 million HIV-related deaths [1]. The most recent estimates for cancer in sub-Saharan Africa suggest in 2012 approximately 591,000 cancer deaths [2]. For people with life-limiting progressive disease, palliative care is advocated by the World Health Organization (WHO) as necessary throughout the disease trajectory, due to the multidimensional (physical, psychological, social and spiritual) problems that are experienced [3-8]. Despite the epidemiology of progressive disease in Africa, and the need to measure multidimensional outcomes for patients and their families, there has been a dearth of evidence of patient and family care needs in both HIV and cancer populations [9,10].

The ability to measure wellbeing among populations across domains of need is essential for effective palliative care. However, the development of appropriate clinical interventions and health systems development is hampered by a lack of locally-generated data. Although some data have been generated on the needs and symptoms of African HIV and cancer populations [5-8], advanced disease populations are rarely investigated using locally well-validated outcome measures. Furthermore, due to the challenges of opioid prescribing and availability in Africa, the palliative care research agenda has been dominated by the study of pain and analgesia [11-16], with less evidence of the needs that constitute the patient holistic experience of illness. This is especially important in developing patient-centred care, as previous studies of patients with advanced disease in sub-Saharan Africa have highlighted the burden of symptoms, the need for information, and the importance attached to spiritual wellbeing [4,5,17,18]. Therefore, the selection of patient-reported outcome measures (PROMs) that are fit for purpose and reflect patient concerns is essential in promoting quality and equity [19]. There are currently few outcome measurement tools that are fit for purpose in African palliative care populations, i.e. tools that reflect the domains relevant to patients in Africa with progressive disease in line with the WHO definition of palliative care [20], and which have been subjected to local validation. It is currently unclear whether the available tools measure the same outcomes and underlying concepts.

This study aimed to measure multidimensional wellbeing, to identify factors associated with patient wellbeing, among patients with advanced HIV and/or cancer in three African countries, and to determine the relationship between two outcome measures validated in this population.

## Method

### Design

This international multicentred study used a cross-sectional self-report design using outcome measures among patients with incurable, progressive disease in South Africa, Uganda and Kenya.

### Setting

South African data were collected at two palliative care facilities of similar size and serving communities in metropolitan areas with a range of socio-economic status, including informal settlements. The first hospice has 10 inpatient beds and serves community patients in the Western Cape Province. The second South African site is a hospice with an eight-bed inpatient unit and a community team serving patients in a metropolitan community in the Eastern Cape Province. Ugandan data were collected at a hospice in the capital city that provides home and day care. Kenyan data were collected from two sites. The first Kenyan site is a hospice in the capital city Nairobi, which cares for patients at different points i.e. at the hospice, home visits, hospital visits and they have a mobile clinic in one of the largest informal settlements within the city environs. The second Kenyan site is a rural hospice situated in the Mount Kenya region in Central Province that operates as a satellite of the capital city hospice, providing holistic care within the hospice, day care services, hospital consultations as well as holding a monthly legal aid clinic. All the participating services aim to provide holistic palliative care in line with the WHO definition [20].

### Recruitment

Inclusion criteria were adult patients (at least 18 years old) with a confirmed HIV and/or cancer diagnosis known to the patient, under palliative care, with sufficient physical and cognitive ability to participate in interviews (as determined by their clinician). Patients were recruited consecutively. All information and consent forms and tools were translated from English (forward and back) into the principal languages of isiXhosa, Afrikaans, Kiswahili, Runyakitara, Luganda, and Kikuyu. Existing FACIT-Pal translations were used where available from the tool provider (i.e., the FACT-G in Afrikaans, Kiswahili) [21]. Informed consent was obtained from all participants. Translation was carried out by the collaborating African research organisations, and crosschecked by staff fluent in both English and the relevant local language. The study was reviewed and approved by the Ethical Review Board of the Hospice Palliative Care Association of South Africa, the Uganda National Council for Science and Technology, and the Kenyan Medical Research Institute.

### Data collection

The following patient demographic and clinical data were collected: age (analysed as three levels 18–39, 40–51 and 52–94), gender, primary diagnosis (HIV or cancer), household size (i.e., number of people in household), number of children for whom the patient is responsible, and primary place of palliative care (home, inpatient/outpatient unit, day care facility). We elected to collect data on the number of children for whom respondents were responsible, rather than the number of biological children given that adults may often care for children other than their own (e.g., grandchildren, nephews and nieces), a situation which has been exacerbated by AIDS-related deaths.

The following tools were administered. Functional status was measured using the ECOG, a very commonly used measure of physical function [22,23]. The African Palliative Care Association African Palliative Outcome Scale (APCA African POS) was used to measure the three-day period prevalence and associated burden of multidimensional problems. This tool was developed across eight sub-Saharan African countries [24] and validated among 682 patients and 437 caregivers [25]. The seven patient-oriented items address pain, symptoms, worry, sharing feelings, feeling life is worthwhile, feeling at peace, and help and advice to plan for the future. The three caregiver-oriented items address family confidence to care, family information and family worry. Each item is scored on a scale of 0–5. A stable three factor structure has been identified [26]. Some item scores were reversed so that for all items and total score, lower score reflects better problem intensity, and the potential score range for patient items is 0–35.

The Functional Assessment of Chronic Illness Therapy-General (FACIT-G) is a 27-item tool that measures the seven-day period prevalence and intensity of problems across four primary quality of life domains: Physical well-being, Social/family well-being, Emotional well-being, and Functional well-being [27]. It has been used and validated in chronic conditions, such as HIV. The additional 19 items of the FACIT-PAL module measure palliative care-related outcomes and are not specific to cancer. The FACIT-G administered with the FACIT-Pal is referred here as the FACIT-G + Pal. The FACIT tools have been globally translated (including into African languages) [28]. A higher score means better status, and the potential score range for FACIT-G + Pal is 0-184 [29].

Research nurses read out the questionnaire items and recorded the patient’s self-report response on their behalf. Self-completion was not used due to potentially limited respondent literacy, and all questionnaires were completed using research nurses to record responses. The selection of a single method (i.e., self-report with research nurse completion) was selected to reduce any potential bias through using a mixture of self-complete and researcher-completion. Research nurses then entered data into a purpose-designed Excel database.

### Analysis

All statistical analyses were completed using IBM-SPSS-19. Means, standard deviations and minimum and maximum scores were calculated for the FACIT-G total score, the POS and FACIT-PAL for Kenya, South Africa and Uganda. Data was analysed according to primary diagnosis, i.e. those with HIV and cancer were analysed as HIV due to cancer being a common presentation among HIV-infected people in Africa. Those analysed as cancer did not have an HIV diagnosis. Separate one-way analyses of variance (ANOVA) were used to examine whether mean scores for FACIT-G, FACIT-Pal and POS were different across the three countries, and MANOVA for the four subscales of FACIT-G due to their high intercorrelation. Because of the large number of statistical comparisons involved, we specified p < 0.01 as the significance level. A multivariate analysis of variance was then conducted for the four subscales of the FACIT-G to test for differences across the three samples. T-tests were used to compare scores on the three measures by gender and diagnosis (HIV, non-HIV). Univariate and multiple regression analysis were used to explore the relative contributions of age (into three equal groups), gender, country, diagnosis and functional status to the dependent variable of FACIT-G score. FACIT-G scores appeared normally distributed. All variables were treated as categorical or dummy variables. Initially each variable was entered individually, with those variables significant at the 5% level entered together into the multivariable model. Finally, FACIT-G subscales and the FACIT-PAL scale were entered into a correlation matrix with the APCA African POS total patient score. The correlation coefficient was Pearson’s r, with the following interpretation: ≤ 0.3 weak, 0.4–0.6 moderate, ≥ 0.7 strong where 0.0 represents no relationship and 1.0 a perfect relationship between two variables [30].

The sample size provided the following: > 95% power to detect a moderate effect size (Cohen’s d ≥ 0.5) at the 0.05 significance level in the t-test analyses; for the correlation analyses this sample provided > 95% power to detect a correlation significantly different from zero at 0.05 significance; for both the univariate and multivariate regression analyses to provide > 95% power to detect a medium size effect (Cohen’s f2 ≥ 0.15) at 0.05 significance.

## Results

### Sample characteristics

In South Africa, Uganda and Kenya respectively, 154, 154 and 153 participants were recruited (total N = 461). The sample characteristics are described in Table 1. In all three countries the majority of the sample was female and had HIV disease.The number of participants with both HIV and cancer (and therefore diagnosed as having a primary HIV diagnosis) was n = 114. Household sizes were similar, although Ugandan respondents were responsible for a greater number of children compared to the Kenyan and South African samples. The Kenyan sample had the best physical function and South Africa the least, and more patients in South Africa were under homecare compared to the other sites. These data reflect the contextual and palliative care model differences between African countries [31].

**Table 1:** Sample characteristics for all three countries (n = 461)

**Country**	**Uganda (n = 154)**	**Kenya (n = 153)**	**South Africa (n = 154)**
**Mean age** (sd, range)	46.25 (sd = 14.39, 19–86)	48.94 (13.02, 18–85)	45.44 (15.67, 19–94)
**Gender** F (%), M (%)	86 (56%), 68 (44%)	101 (66%), 52 (34%)	127 (83%), 26 (17%)
**Primary HIV diagnosis** Yes (%)	114 (74%)	91 (59%)	99 (65%)
**Primary cancer diagnosis** Yes (%)	40 (26%)	62 (41%)	55 (35%)
**Household size i.e., n of people** mean (standard deviation)	5.96 (4.4)	4.27 (2.25)	4.30 (2.49)
**Children** responsible for mean (standard deviation)	4.34 (4.03)	2.78 (2.26)	1.51 (1.54)
**Functional status**			
• Fully active	40 (26%)	73 (48%)	17 (11%)
• Restricted	73 (47%)	46 (30%)	36 (23%)
• Ambulatory	23 (15%)	25 (16%)	34 (22%)
• Limited self care	11 (7%)	7 (5%)	60 (39%)
• Completely disabled	7 (4.5%)	2 (1%)	5 (3%)
• Missing	-	-	1
**Place of care**			
• Home	11 (7%)	1 (1%)	137 (89%)
• Inpatient	7 (4.5%)	-	1
• Day care	45 (29%)	43 (28%)	-
• Outpatient	61 (40%)	109 (71%)	-
• Other	30 (19.5%)	-	14 (9%)
• Missing	-	-	2

### Outcome scores

Table 2 displays the mean, standard deviation and minimum/maximum scores for the FACIT-G Total, FACIT-PAL and APCA African POS for the Ugandan, Kenyan and South African samples and the results of the ANOVAs comparing these across each country. Interestingly, for all three countries the subscale “social and family wellbeing” had the highest (best) score. There was a significant main effect for Country on the FACIT-G Total score and a post-hoc Tukey test showed that this was due to the mean for Uganda being significantly lower (worst) than that in both Kenya and South Africa. Table 2 also presents the mean scores for the Physical, Social/Family, Emotional and Functional Well-Being subscales of the FACIT-G and the results of the MANOVA for the FACIT-G subscales. There was a main effect for Country on Personal, Emotional and Family Well-Being and again post-hoc tests showed that this was explained by the mean values in Uganda being significantly lower (worse) than those in both other countries. Missing data were relatively infrequent across all three questionnaires. There were zero missing data points for the seven items of the African POS. For the 17 items of the Pal subscale of the FACT-Pal, missing data ranged from 2–4 data points. For the 27 FACT-G items, only four items had more than four participants with missing data: these were *I am satisfied with my sex life* (40), *My work (including work at home) is fulfilling* (21), *I feel close to my partner (or person who is my main support)* (13), and *I am able to work (including work at home)* (6).

**Table 2:** Descriptive statistics comparing FACIT-G (total, subscales and ‘Pal’) and APCA African POS across three countries with Cronbach’s α for all Scales

**Country**	**Uganda (n = 154)**	**Kenya (n = 153)**	**South Africa (n = 154)**	**F**	**P=**
	**Mean (s.d., min-max)**	**Mean (s.d., min-max)**	**Mean (s.d., min-max)**		
**FACIT-G subscale: Physical well-being Poss range 0-28**	14.95 (6.84, 0–28) **α = 0.81**	17.63 (8.00, 0–28) **α = 0.86**	19.74 (6.44, 2–28) **α = 0.78**	17.204	0.001
**FACIT-G subscale: Social family well-being Poss range 0-28**	18.78 (7.08, 0–28) **α = 0.77**	22.91 (5.53, 5–28) **α = 0.77**	21.39 (6.12, 3–28) **α = 0.78**	17.101	0.001
**FACIT-G subscale: Emotional well-being Poss range 0-24**	17.45 (6.50, 0–24) **α = 0.83**	18.95 (5.70, 3–25) **α = 0.81**	18.01 (5.62, 4–24) **α = 77**	2.503	0.083
**FACIT-G subscale: Functional well-being Poss range 0-28**	15.75 (7.64, 1–28) **α = 0.86**	19.17 (7.48, 1–28) **α = 0.88**	19.06 (6.48, 1–28) **α = 0.84**	10.951	0.001
**FACIT-G Total Poss range 0-108**	66.93 (21.11, 13–107) **α = 0.90**	78.66 (20.14, 18–108) **α = 0.91**	78.12 (17.05, 20–108) **α = 0.88**	17.635	0.001
**FACIT-Pal subscale Poss range 0-76**	59.69 (11.85, 17–76) **α = 0.81**	62.96 (10.66,22-76 ) **α = 0.83**	60.35 (10.96, 25–76) **α = 0.81**	3.641	0.027
**FACIT-G + Pal Poss range 0-184**	126.63 (31.11, 40–181) **α = 0.92**	141.63 (29.55, 42–184) **α = 0.93**	139.18 (26.41, 45–184) **α = 0.92**	11.62	0.001
**APCA African POS Total Poss range 0-35**	11.93 (6.83, 0–29) **α = 0.72**	11.98 (5.82, 1–26) **α = 0.55**	10.93 (6.55, 0–32) **α = 0.55**	1.312	0.270

Higher FACIT-Pal score means better quality of life, lower POS score means better problem intensity.

Table 3 reports the results of the t-test comparisons for gender and diagnosis, showing there were no significant differences across these variables.

**Table 3:** Results of t-tests comparing gender and primary diagnosis on outcome scores for combined three countries

**Measure**	**Mean (SD)**	**t (df)**	**P =**	
**FACIT-G**	Males (n = 146)	73.12 (20.35)	-1.059 (455)	0.29
	Females (n = 311)	75.26 (20.17)		
**FACIT-Pal**	Males (n = 145)	61.71 (11.38)	0.889 (447)	0.37
	Females (n = 304)	60.70 (11.19)		
**FACIT-G + Pal**	Males (n = 145)	134.92 (30.15)	-.41 (446)	0.68
	Females (n = 303)	136.16 (29.75)		
**APCA African POS**	Males (n = 146)	11.30 (6.45)	-0.662 (458)	0.51
	Females (n = 314)	11.73 (6.40)		
**FACIT-G**	HIV (n = 302)	75.26 (21.51)	1.384 (424)	0.17
	Cancer (n = 124)	72.22 (18.06)		
**FACIT-Pal subscale**	HIV (n = 296)	61.14 (11.31)	0.178 (417)	0.86
	Cancer (n = 123)	60.93 (10.95)		
**FACIT-G + Pal**	HIV (n = 295)	136.55 (31.42)	1.02 (416)	0.31
	Cancer (n = 123	133.24 (27.04)		
**APCA African POS**	HIV (n = 304)	11.70 (6.56)	-0.158 (426)	0.87
	Cancer (n = 124)	11.80 (6.00)		

For the univariate regression analyses, only Country and Functional Status accounted for significant variance in the FACIT-G + Pal, with Functional Status accounting for 13% and Country 5% of variance (see Table 4). In the multiple regression analysis these two variables entered together accounted for 21% of overall variance in FACIT-G + Pal scores (see Table 5). Compared to those with best functional status (i.e., “fully active”) each level of worsening functional status was associated with worsening FACIT-G + Pal score.

**Table 4:** Univariate linear regression analyses: FACIT-G + Pal then POS as the dependent variable.

**Variable**	**Levels**	**Model F**	**R**^**2**^	**p**
**Dependent: FACT-G + Pal**				
Age	3	0.64	0.53	0.00
Gender	2	0.17	0.68	0.00
Country	3	11.62	0.001	0.05
HIV diagnosis	2	1.05	0.31	0.00
Functional status	5	16.55	0.001	0.13
**Dependent: POS**				
Age	3	1.62	0.20	0.01
Gender	2	0.44	0.56	0.00
Country	3	1.31	0.27	0.01
HIV diagnosis	2	0.03	0.87	0.00
Functional status	5	5.15	0.01	0.04

**Table 5:** Multiple regression analyses: FACIT-G + Pal then POS as the dependent variable

**Dependent: FACIT-G + -Pal**							
**Parameter**	**N**	**B**	**Std. error**	**t**	**P value**	**95% Confidence interval for B**	
						**Lower bound**	**Upper bound**
ECOG:							
*Completely disabled*	14	-37.90	7.74	-4.90	.001	-53.12	-22.70
*Limited self-care*	77	-30.68	4.45	-6.90	.001	-39.42	-21.94
*Ambulatory*	82	-22.32	3.99	-5.60	.001	-30.16	-14.48
*Restricted*	154	-21.69	3.33	-6.51	.001	-28.23	-15.14
*Fully active*	130	Ref	Ref	Ref	Ref	Ref	Ref
Country:							
*Uganda*	154	-18.50	3.45	-5.36	.001	-25.27	-11.71
*Kenya*	153	-8.95	3.55	-2.52	.01	-15.94	-1.97
*South Africa* (*reference group)*	150	Ref	Ref	Ref	Ref	Ref	Ref
**Dependent: POS**							
	**N**	**B**	**Std. error**	**t**	**P value**	**95% Confidence interval for B**	
						**Lower bound**	**Upper bound**
**ECOG:**							
***Completely disabled***	14	3.85	1.77	2.16	0.03	0.35	7.34
***Limited self-care***	77	4.64	1.00	4.61	0.00	2.67	6.63
***Ambulatory***	82	3.95	0.91	4.36	1	2.17	5.74
***Restricted***	154	3.20	0.76	4.20	0.00	1.70	4.70
***Fully active (reference group)***	130	Ref	Ref	Ref	1 0.00 1 Ref	Ref	Ref
**Country:**							
***Uganda***	154	1.98	0.77	2.55	0.01	0.45	3.51
***Kenya***	153	2.78	0.83	3.46	0.00	1.20	4.36
***South Africa *** **(*reference group)***	150	Ref	Ref	Ref	1 Ref	Ref	Ref

For the univariate regression analyses with POS as dependent variable (see Table 4), only functional status was significant (accounting for 4% of variance), although we also retained country in the multivariable model due to earlier evidence of country effect. In the final multivariable model (see Table 5, 7% of variance accounted for), compared to those with best functional status (i.e. “fully active”) each level of worsening functional status was associated with worse POS score. Compared to South Africa, Ugandan participants had a worse score, and Kenyans had a slightly poorer score.

The matrix of correlational coefficients (see Table 6) revealed a moderate correlation between the APCA African POS and the FACIT-Pal (0.60), the FACIT-G and the APCA African POS (0.64), and the FACIT-G + Pal with the POS (0.66).

**Table 6:** Correlations between FACIT-G and Pal total and subscales with APCA African POS

**Scale/Subscale**		**FACIT-G**	**Personal well-being**	**Social well-being**	**Emotional well-being**	**Family well-being**	**FACIT-Pal subscale**	**POS**
Personal well-being	r =	.809						
	p <	.000						
	n =	458						
Social well-being	r =	.577	.207					
	p <	.000	.000					
	n =	458	460					
Emotional well-being	r =	.727	.482	.259				
	p <	.000	.000	.000				
	n =	458	459	459				
Family well-being	r =	.836	.652	.290	.478			
	p <	.000	.000	.000	.000			
	n =	458	458	458	458			
FACIT-Pal subscale	r =	.774	.589	.470	.602	.641		
	p <	.000	.000	.000	.000	.000		
	n =	449	449	449	449	450		
APCA African POS	r =	-.636	-.574	-.315	-.517	-.472	-.605	
	p <	.000	.000	.000	.000	.000	.000	
	n =	458	460	460	459	459	450	
FACIT-G + Pal	r =	.971	.777	.570	.772	.810	.903	-0.663
Total score	p <	.000	.000	.000	.000	.000	.000	.000
	n=	449.	449	449	449	449	449	449

## Discussion

These data are the first to provide self-report data using measures of multi-dimensional wellbeing measures specifically designed for patients with progressive illness across diagnoses, and was conducted in three African countries.

Our samples were relatively young which reflects the HIV population, and are therefore responsible for children. This has important implications for palliative care provision, as hospice and palliative care aims to provide family-based support. They also had relatively high physical function, which reflects the African model of palliative care integration throughout the HIV disease trajectory [9].

With respect to the FACIT-Pal subscales, scores appear to be best for the social/family wellbeing dimension. This reflects both the data on number of children patients were responsible for, but may also reflect African cultures which are more communal and less individualistic than Western societies, therefore support may have been accessed in the community. Poverty also appears to be an important factor in our sample, as the Ugandan sample had worse scores on most outcomes than respondents in Kenya and South Africa, and Uganda has a lower gross domestic product (GDP) than the other countries. Importantly, no difference in total scores was found when comparing HIV and cancer patients, which suggests that services can approach both populations with a similar care and thus avoid the “silo” approach of providing separate oncology and HIV palliative care services. Importantly, physical function was strongly associated with worse status for both outcome measures (FACT-G + Pal and POS). The role of rehabilitation is key for patients with progressive illness, as function can be maximised within palliative care planning [32,33]. The worse total scores for Ugandan patients in multivariable analysis may also reflect the referral criteria across countries, in that the Ugandan service may assume care for those with greatest need.

In terms of the comparison of measures we note a statistically significant, moderate correlation between the POS and APCA African POS/FACIT dimensions, suggesting that these two tools measure related but conceptually distinct aspects of wellbeing. The FACT-G + Pal is slightly more strongly correlated to the POS that the Pal dimension alone, suggesting that the FACT-G also supports the measurement of outcomes for advanced disease, and that the Pal should be used with FACT-G and not alone.

There are a number of limitations to our study. Firstly we note that the APCA African POS is the only measure that has been fully validated for use with palliative care populations in sub-Saharan Africa, although the FACT-Pal has undergone rigorous translation. We also assume equivalence between the tools in the different languages, although there may be conceptual differences related to language and culture. There may be a sampling bias that have led to an underestimation of problems, as palliative care provision is not yet fully integrated into health systems [34], and therefore those who have not entered hospice or palliative care may have worse problems. Lastly our cross sectional design can identify associations but not causality.

## Conclusions

The care of those with life limiting, progressive illness within low and middle income settings requires appropriate care to ensure optimal quality of life, minimal suffering and a good death. Our data reveal across all countries that family and social wellbeing were the worst problems, which is consistent with cultures with more communal rather than individualistic characteristics. Importantly, total self-reported quality of life and palliative care problem severity does not differ by diagnosis, suggesting that separate palliative care services are not needed for each population, although of course there may be variation between diagnoses by item. There is a strong requirement for clinicians to ensure that assessment and models of care address the social dimensions of wellbeing. The clinical presentation of problems may differ according to country, with providers facing higher levels of patient need in economically poorer settings. However, a common pattern of worsening wellbeing was found with declining physical function, and maximising function within a clinical approach of palliation is essential to enhance patient outcomes.

## Competing interests

The authors have no competing interests.

## Authors’ contributions

RH conceived the study, RS led analysis, all authors had oversight and implementation responsibility and all commented on and approved the manuscript.
